# Sex interacts with age-dependent change in the abundance of lice-infesting Amur Falcons (*Falco amurensis*)

**DOI:** 10.1007/s00436-020-06753-w

**Published:** 2020-06-18

**Authors:** Imre Sándor Piross, Manju Siliwal, R. Suresh Kumar, Péter Palatitz, Szabolcs Solt, Péter Borbáth, Nóra Vili, Nóra Magonyi, Zoltán Vas, Lajos Rózsa, Andrea Harnos, Péter Fehérvári

**Affiliations:** 1 Department of Biomathematics and Informatics, University of Veterinary Medicine, Budapest, Hungary; 2 Balaton Limnological Institute, Centre for Ecological Research, Tihany, Hungary; 3 Department of Animal Ecology and Conservation Biology, Wildlife Institute of India, Dehradun, India; 4 Department of Endangered Species Management, Wildlife Institute of India, Dehradun, India; 5 MME BirdLife Hungary, Red-footed Falcon Workgroup, Budapest, Hungary; 6 Bükk National Park Directorate, Eger, Hungary; 7 Conservation Genetics Research Group, Department of Ecology, Institute for Biology, University of Veterinary Medicine, Budapest, Hungary; 8 Doctoral School of Biology and Sportbiology, Faculty of Sciences, University of Pécs, Pécs, Hungary; 9 Hungarian Natural History Museum, Budapest, Hungary; 10 GINOP Evolutionary Systems Research Group, Institute of Evolution, Centre for Ecological Research, Tihany, Hungary

**Keywords:** Louse, Falconidae, Sex-biassed infestation, Phthiraptera, Amblycera, Ischnocera

## Abstract

Sex-biassed and age-biassed parasite infections are common in nature, including ectoparasites-vertebrate host systems. We investigated the effect of Amur Falcons’ sex, age and body size on the abundance of their lice at a migratory stopover site, where the falcons’ habitat use and behaviour are more homogeneous across sex and age categories than during the breeding season. We sampled Amur Falcons in Nagaland, India at major roosting sites in 2016. We applied generalized linear models (with negative binomial distribution and log-link) to model the abundance of their two most numerous lice (*Colpocephalum subzerafae* and *Degeeriella rufa*) using the host age category (juvenile or adult) and wing length, both in interaction with sex, as explanatory variables. The abundance of *C. subzerafae* was only affected by host age, being nearly four times higher on juveniles than on adults. Juveniles were also more infested with *D. rufa* than the adults. Additionally, the abundance of the latter species was lower on adult male Falcons as compared to adult females. A juvenile bias in ectoparasite infestations is common in nature, probably due to juveniles being immunologically naïve, more resource-limited and may be inexperienced in body maintenance behaviours like preening and grooming. On the other hand, female-biassed infestations are much rarer than male-biassed infestations. We briefly discuss the possible causes of female-biassed infestations on Amur Falcons reported here, and in the closely related Red-footed Falcon and Lesser Kestrel as reported in the literature.

**Electronic supplementary material:**

The online version of this article (10.1007/s00436-020-06753-w) contains supplementary material, which is available to authorized users.

## Introduction

Though avian ectoparasites rarely have detrimental effects on their hosts, levels of their infestations still covaries with host health status for two reasons. First, birds with poor body condition may invest fewer resources into antiparasitic defences, thus allowing more parasites to infest, survive and multiply on their bodies. Second, the rise of ectoparasite infestations exerts an increasing metabolic cost on the hosts. Thus, high parasite burdens could constitute both a cause and a consequence of poor health status in birds (Clayton et al. 2015).

This is particularly true for large-bodied bird species like raptors because they tend to host relatively high ectoparasite burdens (Rózsa 1997). Further, since several populations have declined dramatically, many raptors are considered vulnerable to extinction. Thus, the importance of monitoring the health status of their populations, as judged from levels of parasite infestations, is increasingly important for conservation purposes (Órdenes et al. 2005; Liébana et al. 2011; Saxena 2017; Tinajero et al. 2019; Yosef et al. 2019). Moreover, avian ectoparasites may also be threatened by extinction and constitute conservation values themselves (Rózsa and Vas 2015; Dougherty et al. 2016; Bulgarella and Palma 2017; Kwak 2018; Kwak et al. 2019; West et al. 2019). Establishing baseline values for parasite infections in natural populations must necessarily be controlled for the most important factors affecting individual ectoparasite loads. Most notably, host age, sex and body size likely affects individual infestation levels, and the seasonality of infestation dynamics must also be taken into account (Lamb and Galloway 2016; Yunik et al. 2016).

Observational studies, like our present one, have two main problems. First, they cannot unveil the direction of causality due to the researchers’ lack of control over the independent variables. Second, it is a matter of more-or-less subjective decisions which independent variables are to be measured and analysed. To partially relieve these problems, it is favourable to choose model systems where at least some independent variables show little or no variation in the population.

Amur Falcons (*Falco amurensis* Radde, 1863) breed in East Asia (Transbaikalia, Amurland, North-Eastern China) and winter in southern Africa making their migratory route the longest among raptors. They form huge, high-density aggregations at communal roosting sites in northeast India during post-nuptial migration, where hundreds of thousands of birds can gather. At this stage of their life cycle, Amur Falcons constitute exemplary subjects of parasite ecological studies for the following reasons. First, parasite transmission is enhanced by their nocturnal behaviour leading to close body proximity. Therefore, it is reasonable to assume that the individuals’ observed louse load depends mostly on their actual resistance and less on their individual history, whether they had contracted lice formerly or not. Second, unlike during the breeding season, the different sexes and age groups live a similar way of life at these stopover sites.

In the current study, we aimed to utilize this unique opportunity to investigate how the abundance of lice depends on the host’s body size, sex and age during the Amur Falcons’ autumn migratory stopover period at their large Indian roosting sites.

## Materials and methods

### Study site

Data collection took place in Nagaland, India (N25.67, E94.11) where Amur Falcons were trapped and ringed within the framework of an ongoing research project led by Wildlife Institute of India (WII). Birds were mist-netted at three different roost sites in November of 2016. The three sites were located approximately 50 km from each other, two of them were located in secondary sub-tropical evergreen rainforests and the third one by a teak tree plantation. Two sites each had over 100,000 individuals at the time of trapping, while one site was utilized by over 15,000 roosting individuals.

### Data collection

We ringed and recorded age and sex of all the 50 Amur Falcons trapped. Only juveniles (i.e. first calendar year birds) and adult (at least third calendar year) birds were selected for the analyses. We recorded wing length, as a proxy of body size that is easy to measure under field conditions. Blood samples were also collected by puncturing the brachial vein of the wing, and subsequently stored in 70% ethanol. These samples were later used to identify the sex of juvenile birds.

Dust-ruffling (Clayton and Drown 2001) was used to remove lice from hosts. The plumage was treated with pyrethrin powder over a white tray. Lice falling off were collected into a centrifuge tube containing 70% ethanol. After 5 min, the plumage was gently ruffled to dislodge the remaining parasites. Ectoparasite sampling was carried out solely by P.F. The identification of lice was carried out by M.S. and R.S.K. at the Wildlife Institute of India (WII) using a stereoscopic microscope based on Price et al. (2003).

### Molecular sexing of juveniles

The molecular sexing of the juveniles was carried out at the WII by M.S. and R.S.K. Total genomic DNA was extracted from the collected blood samples using Qiagen® DNeasy Blood & Tissue Kit (Quiagen Valencia CA.). Sex was determined by amplifying the CHD1-W and CHD1-Z gene introns, using the 2550F and 2718R primer pair (Fridolfsson and Ellegren 1999). To verify the molecular sexing results, two methods were used: first, another intronic part of the CHD1 gene was amplified in parallel using the primer pair (CHD1-i16F and CHD1-i16R; Suh et al. 2011) in a subset of samples (*N* = 10). Second, 18 adult birds with known sex were additionally analysed. Both primer pairs gave congruent results, and sex determined by molecular analysis agreed with adult phenotypic sex. PCR reactions were performed using the conditions as described by the authors publishing the primers. PCR products were evaluated by agarose gel-electrophoresis.

### Statistical methods

We applied generalized linear models (GLM) with negative binomial error distribution and log-link (Zuur et al. 2009) to model the mean abundances of the two common louse species, *Colpocephalum subzerafae* Tendeiro, 1988b and *Degeeriella rufa* Burmeister, 1838. Since the sample size only allows us to investigate the effect of a handful of explanatory variables, we chose to incorporate the biologically most relevant potential predictors into our initial model. These were the following: the bird’s age class (juvenile or adult), their wing length (mm) as a proxy of body size, and these variables’ interaction with sex. We chose not to incorporate the roosting localities into our models since the birds change their nocturnal roosting sites frequently within the season, and we saw no relevant differences between the sites from this study’s point of view. We used likelihood-ratio tests for model selection. We removed explanatory variables from our initial models, and if this caused the model to fit significantly worse (*ɑ* = 0.05), we kept the variable in the model. Our initial models contained sex, wing length (mm) and their interaction, age (juvenile or adult) and its interaction with sex. We centred the wing length variable (subtracted the mean) to stabilize model fit. For all analyses and for preparing the figures, we used R 3.6.1 (R Core Team 2019) and the ggplot2 3.2.0 (Wickham 2016), glmmTMB 0.2.3 (Brooks et al. 2017), gridExtra 2.3 (Auguie 2017), lsmeans 2.30–0 (Lenth 2016) and the RcmdrMisc 2.5-1 (Fox 2018) packages.

## Results

We found three louse species on the Amur Falcons, all of which have been described from this host previously (Price et al. 2003; Piross et al. 2015). *Laemobothrion (Laemobothrion) tinnunculi* Linnaeus, 1758 was scarcely found on birds with only five hosts infested and each one carried only a single louse individual. Contrarily, both *Colpocephalum subzerafae* and *Degeeriella rufa* were abundant on birds, thus providing large enough samples for statistical analyses. The descriptive statistics (Reiczigel et al. 2019) of their infestation are provided in Table 1.

**Table 1:** Descriptive statistics of the louse infestation of the Amur Falcons (*Falco amurensis*) by age, louse species and sex (SD: standard deviation)

Louse species	Age	Sex	Infected	Hosts	Prevalence	Mean abundance ± SD	Median abundance	Mean intensity ± SD	Median intensity	Variance/mean
*C. subzerafae*	Juveniles	Male	15	20	75	6 ± 6.8	4	8.1 ± 6.7	4	7.5
		Female	10	11	91	8.9 ± 7.9	8	9.8 ± 7.7	8	7.0
		All	25	31	81	7.1 ± 7.2	4	8.8 ± 7	7	7.3
	Adults	Male	6	10	60	1.8 ± 2.1	1	3 ± 1.9	3	2.4
		Female	6	9	67	1.9 ± 1.7	2	2.8 ± 1.2	3	1.5
		All	12	19	63	1.8 ± 1.9	1	2.9 ± 1.5	3	1.9
*D. rufa*	Juveniles	Male	20	20	100	9.4 ± 5.1	9	9.4 ± 5.1	9	2.8
		Female	11	11	100	10.4 ± 4.5	9	10.4 ± 4.5	9	1.9
		All	31	31	100	9.7 ± 4.8	9	9.7 ± 4.8	9	2.4
	Adults	Male	6	10	60	1.8 ± 2.5	1	3 ± 2.7	2	3.6
		Female	9	9	100	4.9 ± 4.8	3	4.9 ± 4.8	3	4.7
		All	15	19	79	3.3 ± 4	2	4.1 ± 4.1	2	4.9

**Fig. 1:**
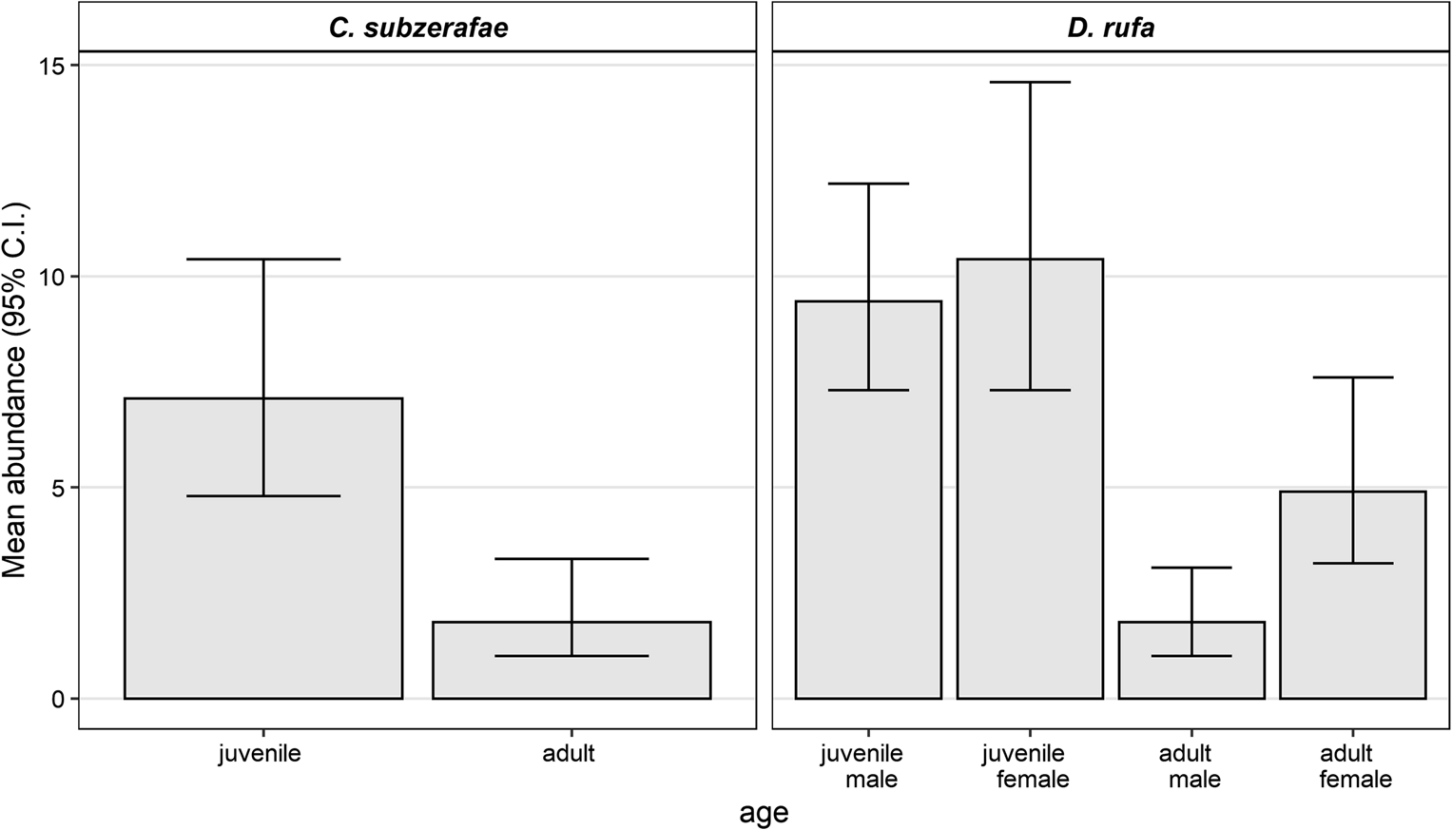
Results of the GLMs modelling the mean abundance of the louse species on the Amur Falcons (*Falco amurensis*). The mean abundance of *Colpocephalum subzerafae* is higher on juveniles than on males. In the case of *Degeeriella rufa*, we found interaction between the sex and the age of the birds. The mean abundance of *D. rufa* is similarly high among juveniles for both sexes, while it is higher for adult females than adult males

Our models indicated that only age exerted a significant effect (*p* = 0.0006) on the mean abundance of *C. subzerafae* (see the Supplementary Material). The mean abundance on adults was 1.8 (95% C.I. 1.0–3.3) compared to 7.1 (95% C.I. 4.8–10.4) on juveniles (see Table 2 and Fig. 1).

**Table 2:** Abundances (and their 95% C.I.) of the different louse species on Amur Falcons (*Falco amurensis*) predicted by the GLMs

Louse species	Age	Sex	Abundance estimate	95% C.I.	
*C. subzerafae*	Juvenile		7.1	4.8	10.4
	Adult		1.8	1.0	3.3
*D. rufa*	Juvenile	Male	9.4	7.3	12.2
		Female	10.4	7.3	14.6
	Adult	Male	1.8	1.0	3.1
		Female	4.9	3.2	7.6

In case of *D. rufa*, on the other hand, sex, age and their interaction exhibited a significant effect (*p* = 0.0006) on mean abundance (see the Supplementary Material). The mean abundance was 9.4 (95% C.I. 7.3–12.2) on juvenile males and 10.4 (95% C.I. 7.3–14.6) on juvenile females. In the case of adults, the abundances were lower and differed between the two sexes. Adult females showed a higher mean of *D. rufa* abundance (4.9, 95% C.I. 3.2–7.6) than males (1.8, 95% C.I. 1.0–3.1; see Table 2.). The results of the likelihood ratio tests and the AIC and BIC values of the models are provided in the Supplementary Material.

## Discussion

There is a growing body of information concerning the environmental factors influencing the distribution and abundance of lice in comparisons across different bird species (Rózsa 1997; Moyer et al. 2002; Galloway and Lamb 2017; Lamb and Galloway 2019). Contrarily, it is much less understood how host individual factors (like age, body size and their interaction with sex) influence the distribution of lice within a particular bird population (but see Palma et al. 2002; Szczykutowicz et al. 2006; Durkin et al. 2015; Leonardi and Quintana 2017; Leonardi et al. 2018).

We have collected lice from Amur Falcons sampled in Nagaland, India at a migratory stopover site, where these birds gather to form huge autumn roosting flocks before crossing the Indian subcontinent and the Arabian Sea towards their African wintering grounds. Our aim was to investigate the effect of falcon sex, age and body size on the abundance of their two most abundant species of lice. We applied generalized linear models to model parasite abundance using host age, wing length and their interaction with sex as explanatory variables.

We demonstrated that the age class (juvenile vs. adult) of Amur Falcons covaries with the abundance of both *C. subzerafae* and *D. rufa* so that juveniles were more infested than adults. Juvenile-biassed levels of ectoparasite infestation have been frequently reported by other authors (Potti and Merino 1995; Hamstra and Badyaev 2009; Rivera-Parra et al. 2014), probably due to several reasons. First, juveniles are immunologically naïve, and the amblyceran lice (including *C. subzerafae*) partially feed on living tissues and, thus, interact with the host immune system (Møller and Rózsa 2005). Second, young birds may not be able to allocate as much time to body maintenance behaviours like preening (birds’ primary defence mechanism against lice) as the adult birds. Finally, the efficacy of preening may well depend on individual practice, even though the effect of experience could not be experimentally verified in domestic Rock Pigeons (*Columba livia* Gmelin, 1789; Villa et al. 2016).

The results presented here indicate that *D. rufa* also exhibits female-biassed infestation during the autumn period at a migratory stopover site, where the falcons’ habitat usage and behaviour are presumed to be uniform across sexes. Female-biassed infestations are not unprecedented; Ortego et al. (2007) showed that the *D. rufa* infestations in Lesser Kestrels (*Falco naumanni* Fleischer, 1818) tend to be female-biassed. However, it is far more often the male sex that hosts higher levels of infection (Poulin 1996; Zuk and McKean 1996; Morales-Montor et al. 2004). For example, male-biassed louse infestations were found among White-throated Dippers (*Cinclus cinclus* (Linnaeus, 1758); Doyle et al. 2005) and various seabirds (Rivera-Parra et al. 2014).

The causes of female bias observed here are not known, but several possible reasons can be put forward to explain this phenomenon:

First, one could hypothesise that females constitute more optimal hosts for these lice due to their somewhat larger body size. This seems unlikely since we have statistically controlled for body size (using wing length as a proxy for it) in the present study, and also in our other study on Red-footed Falcons (*Falco vespertinus* Linnaeus, 1766; Piross et al. 2020).

Second, Red-footed Falcons — previously considered conspecific with Amur Falcons — also exhibit female-biassed infestations by the same two louse species examined here, at least during the early days of the breeding period (Piross et al. 2020). During the breeding season, the female-biassed infestations might be driven by a difference in how sexes allocate their time; females may allocate less time to body maintenance. The Amur falcons’ female-biassed infestations with *D. rufa* during the autumn migration could simply be a remnant of a previous infestation difference that built up during the summer breeding season. However, this does not explain why only *D. rufa* showed female-biassed infestation. A notable difference between the two species is that they evade preening differently. *Degeeriella rufa* similarly to some other ischnocerans attaches itself to feathers. Amblycerans, on the other hand, tend to use more active evasion techniques and different refugia. *Colpocephalum* species, for example, may hide inside of feather shafts (Clayton and Johnson 2003). Assuming that there is a difference here in the efficacy of the two broad evasion strategies, we hypothesise that the females may be able to reduce the number of *C. subzerafae* more quickly; however, this explanation lacks empirical evidence.

Lastly, female-biassed parasitism can be attributed to sexual dichromatism. Adult male Amur Falcons and Red-footed Falcons display a dark bluish-grey colouration based on melanin pigments. Since melanin is known to make feathers more resistant to mechanical abrasion (Bonser 1995), dark feathers may be harder to chew for lice. Contrary to this expectation, however, Bush et al. (2006) showed that dark colouration in rock pigeons provided no defence against the pigeon wing lice, *Columbicola columbae* (Linnaeus 1758). However, the ischnoceran wing lice like *C. columbae* and *D. rufa* graze the barbules of the down feathers that tend to be unpigmented, but they do not feed on the heavily pigmented, interlocked barbs of the vane of cover feathers (Clayton et al. 2015). Therefore, the difference in the melanin pigmentation between the sexes is unlikely to cause the infestation differences we documented.

Overall, we conclude that birds can show age- and sex-biassed levels of louse infestation even during migration, when birds of different age and sex live an apparently similar way of life. Our study highlights the importance of following the seasonal changes of sex-biassed parasite infections through the whole life cycle of the host to develop a better understanding of host-parasite systems.

## Electronic supplementary material

ESM 1 (DOCX 44.9 kb)
