# Supplementary material for: Sex interacts with age-dependent change in the abundance of lice-infesting Amur Falcons (Falco amurensis)
Source: Parasitol Res. 2020 Jun 18;119(8):2579–85. doi: 10.1007/s00436-020-06753-w (PMC7366564; doi:10.1007/s00436-020-06753-w)
Supplement: Supplementary file 1 — (DOCX 44.9 kb) [file 436_2020_6753_MOESM1_ESM.docx]

Sex interacts with age-dependent change in the abundance of lice infesting Amur Falcons (*Falco amurensis*)

Imre Sándor Piross^1,2^, Manju Siliwal^3^, R. Suresh Kumar^4^, Péter Palatitz^5^, Szabolcs Solt^5^, Péter Borbáth^6^, Nóra Vili^7^, Nóra Magonyi^7,8^, Zoltán Vas^9^, Lajos Rózsa^10^, Andrea Harnos^1^, Péter Fehérvári^1*^

^*^Corresponding author. fehervari.peter@univet.hu, telephone: +36709469331

1: Department of Biomathematics and Informatics, University of Veterinary Medicine, Budapest, Hungary

2: Balaton Limnological Institute, Centre for Ecological Research, Tihany, Hungary

3: Department of Animal Ecology and Conservation Biology, Wildlife Institute of India, Dehradun, India

4: Department of Endangered Species Management, Wildlife Institute of India, Dehradun, India

5: MME BirdLife Hungary, Red-footed Falcon Workgroup, Budapest, Hungary

6: Bükk National Park Directorate

7: Conservation Genetics Research Group, Department of Ecology, Institute for Biology, University of Veterinary Medicine, Budapest, Hungary

8: Doctoral School of Biology and Sportbiology, Faculty of Sciences, University of Pécs, Pécs, Hungary

9: Hungarian Natural History Museum, Budapest, Hungary

10: GINOP Evolutionary Systems Research Group, Institute of Evolution, Centre for Ecological Research

Author ORCID iD

Imre Sándor Piross 0000-0003-1201-5085

R. Suresh Kumar

Manju Siliwal 0000-0002-7037-7095

Péter Palatitz 0000-0002-7472-1308

Szabolcs Solt 0000-0002-1008-1698

Péter Borbáth

Nóra Vili 0000-0001-7241-231X

Nóra Magonyi

Zoltán Vas 0000-0002-1361-180X

Lajos Rózsa 0000-0002-9470-0934

Andrea Harnos

Péter Fehérvári

# Abstract

Sex-biased and age-biased parasite infections are common in nature, including ectoparasites-vertebrate host systems. We investigated the effect of Amur Falcons’ sex, age and body size on the abundance of their lice at a migratory stopover site, where the falcons’ habitat use, and behaviour are more homogeneous across sex and age categories than during the breeding season.

We sampled Amur Falcons in Nagaland, India, at major roosting sites in 2016. We applied generalised linear models (with negative binomial distribution and log-link) to model the abundance of their two most numerous lice (*Colpocephalum subzerafae* and *Degeeriella rufa*) using the host age category (juvenile or adult) and wing length, both in interaction with sex, as explanatory variables.

The abundance of *C. subzerafae* was only affected by host age, being nearly four times higher on juveniles than on adults. Juveniles were also more infested with *D. rufa* than the adults. Additionally, the abundance of the latter species was lower on adult male Falcons as compared to adult females.

A juvenile bias in ectoparasite infestations is common in nature, probably due to juveniles being immunologically naïve, more resource-limited, and may be inexperienced in body maintenance behaviours like preening and grooming. On the other hand, female-biased infestations are much rarer than male-biased infestations. We briefly discuss the possible causes of female-biased infestations on Amur Falcons reported here, and in the closely related Red-footed Falcon and Lesser Kestrel as reported in the literature.

## Keywords

louse, Falconidae, sex-biased infestation, Phthiraptera, Amblycera, Ischnocera

# Introduction

Though avian ectoparasites rarely have detrimental effects on their hosts, levels of their infestations still covaries with host health status for two reasons. First, birds with poor body condition may invest fewer resources into antiparasitic defenses, thus allowing more parasites to infest, survive and multiply on their bodies. Second, the rise of ectoparasite infestations exerts an increasing metabolic cost on the hosts. Thus, high parasite burdens could constitute both a cause and a consequence of poor health status in birds (Clayton et al. 2015).

This is particularly true for large-bodied bird species like raptors, because they tend to host relatively high ectoparasite burdens (Rózsa 1997). Further, since several populations have declined dramatically, many raptors are considered vulnerable to extinction. Thus, the importance of monitoring the health status of their populations, as judged from levels of parasite infestations, is increasingly important for conservation purposes (Órdenes et al. 2005; Liébana et al. 2011; Saxena 2017; Tinajero et al. 2019; Yosef et al. 2019). Moreover, avian ectoparasites may also be threatened by extinction and constitute conservation values themselves (Rózsa and Vas 2015; Dougherty et al. 2016; Bulgarella and Palma 2017; Kwak 2018; Kwak et al. 2019; West et al. 2019). Establishing baseline values for parasite infections in natural populations must necessarily be controlled for the most important factors affecting individual ectoparasite loads. Most notably, host age, sex, and body size likely affects individual infestation levels, and the seasonality of infestation dynamics must also be taken into account (Lamb and Galloway 2016; Yunik et al. 2016).

Observational studies, like our present one, have two main problems. First, they cannot unveil the direction of causality due to the researchers’ lack of control over the independent variables. Second, it is a matter of more-or-less subjective decisions which independent variables are to be measured and analysed. To partially relieve these problems, it is favourable to choose model systems where at least some independent variables show little or no variation in the population.

Amur Falcons (*Falco amurensis* Radde, 1863) breed in East Asia (Transbaikalia, Amurland, North-Eastern China) and winter in southern Africa making their migratory route the longest among raptors. They form huge, high-density aggregations at communal roosting sites in northeast India during post-nuptial migration, where hundreds of thousands of birds can gather. At this stage of their life cycle, Amur Falcons constitute exemplary subjects of parasite ecological studies for the following reasons. First, parasite transmission is enhanced by their nocturnal behaviour leading to close body proximity. Therefore, it is reasonable to assume that the individuals’ observed louse load depends mostly on their actual resistance and less on their individual history; whether they had contracted lice formerly or not. Second, unlike during the breeding season, the different sexes and age groups live a similar way of life at these stopover sites.

In the current study, we aimed to utilise this unique opportunity to investigate how the abundance of lice depends on the host’s body size, sex and age during the Amur Falcons’ autumn migratory stopover period at their large Indian roosting sites.

# Materials and methods

## Study site

Data collection took place in Nagaland, India (N25.67, E94.11), where Amur Falcons were trapped and ringed within the framework of an ongoing research project led by Wildlife Institute of India (WII). Birds were mist netted at three different roost sites in November of 2016. The three sites were located approximately 50 km from each other, two of them were located in secondary sub-tropical evergreen rainforests and the third one by a teak tree plantation. Two sites each had over 100 000 individuals at the time of trapping, while one site was utilised by over 15 000 roosting individuals.

## Data collection

We ringed and recorded age and sex of all the 50 Amur Falcons trapped. Only juveniles (i.e. 1^st^ calendar year birds) and adult (at least 3^rd^ calendar year) birds were selected for the analyses. We recorded wing length, as a proxy of body size that is easy to measure under field conditions. Blood samples were also collected by puncturing the brachial vein of the wing, and subsequently stored in 70% ethanol. These samples were later used to identify the sex of juvenile birds.

Dust-ruffling (Clayton and Drown 2001) was used to remove lice from hosts. The plumage was treated with pyrethrin powder over a white tray. Lice falling off were collected into a centrifuge tube containing 70% ethanol. After five minutes, the plumage was gently ruffled to dislodge the remaining parasites. Ectoparasite sampling was carried out solely by P.F. The identification of lice was carried out by M.S. and R.S.K. at the Wildlife Institute of India (WII) using a stereoscopic microscope based on Price et al. (2003).

## Molecular sexing of juveniles

The molecular sexing of the juveniles was carried out at the WII by M.S. and R.S.K. Total genomic DNA was extracted from the collected blood samples using Qiagen® DNeasy Blood & Tissue Kit (Quiagen Valencia CA.). Sex was determined by amplifying the CHD1-W and CHD1-Z gene introns, using the 2550F and 2718R primer pair (Fridolfsson and Ellegren 1999). To verify the molecular sexing results, two methods were used: first, another intronic part of the CHD1 gene was amplified in parallel using the primer pair (CHD1-i16F and CHD1-i16R; Suh et al. 2011) in a subset of samples (N=10). Second, 18 adult birds with known sex were additionally analysed. Both primer pairs gave congruent results, and sex determined by molecular analysis agreed with adult phenotypic sex. PCR reactions were performed using the conditions as described by the authors publishing the primers. PCR products were evaluated by agarose gel-electrophoresis.

## Statistical methods

We applied generalised linear models (GLM) with negative binomial error distribution and log-link (Zuur et al. 2009) to model the mean abundances of the two common louse species, *Colpocephalum subzerafae* Tendeiro, 1988b and *Degeeriella rufa* Burmeister, 1838. Since the sample size only allows us to investigate the effect of a handful of explanatory variables, we chose to incorporate the biologically most relevant potential predictors into our initial model. These were the following: the bird’s age class (juvenile or adult), their wing length (mm) as a proxy of body size, and these variables’ interaction with sex. We chose not to incorporate the roosting localities into our models since the birds change their nocturnal roosting sites frequently within the season, and we saw no relevant differences between the sites from this study’s point of view. We used likelihood-ratio tests for model selection. We removed explanatory variables from our initial models, and if this caused the model to fit significantly worse (ɑ=0.05), we kept the variable in the model. Our initial models contained sex, wing length (mm) and their interaction, age (juvenile or adult) and its interaction with sex. We centred the wing length variable (subtracted the mean) to stabilise model fit. For all analyses and for preparing the figures we used R 3.6.1 (R Core Team 2019) and the ggplot2 3.2.0 (Wickham 2016), glmmTMB 0.2.3 (Brooks et al. 2017), gridExtra 2.3 (Auguie 2017), lsmeans 2.30-0 (Lenth 2016) and the RcmdrMisc 2.5-1 (Fox 2018) packages.

# Results

We found three louse species on the Amur Falcons, all of which have been described from this host previously (Price et al. 2003; Piross et al. 2015). *Laemobothrion (Laemobothrion) tinnunculi* Linnaeus, 1758 was scarcely found on birds with only five hosts infested and each one carried only a single louse individual. Contrarily, both *Colpocephalum subzerafae* and *Degeeriella rufa* were abundant on birds, thus providing large enough samples for statistical analyses. The descriptive statistics (Reiczigel et al. 2019) of their infestation are provided in Table 1.

Our models indicated that only age exerted a significant effect (p=0.0006) on the mean abundance of *C. subzerafae* (see the Supplementary Material). The mean abundance on adults was 1.8 (95% C.I. 1.0–3.3) compared to 7.1 (95% C.I. 4.8–10.4) on juveniles (see Table 2 and Figure 1).

In case of *D. rufa*, on the other hand, sex, age, and their interaction exhibited a significant effect (p=0.0006) on mean abundance (see the Supplementary Material). The mean abundance was 9.4 (95% C.I. 7.3–12.2) on juvenile males and 10.4 (95% C.I. 7.3–14.6) on juvenile females. In the case of adults, the abundances were lower and differed between the two sexes. Adult females showed a higher mean of *D. rufa* abundance (4.9, 95% C.I. 3.2–7.6) than males (1.8, 95% C.I. 1.0–3.1; see Table 2.). The results of the likelihood ratio tests and the AIC and BIC values of the models are provided in the Supplementary Material.

# Discussion

There is a growing body of information concerning the environmental factors influencing the distribution and abundance of lice in comparisons across different bird species (Rózsa 1997; Moyer et al. 2002; Galloway and Lamb 2017; Lamb and Galloway 2019). Contrarily, it is much less understood how host individual factors (like age, body size and their interaction with sex) influence the distribution of lice within a particular bird population (but see Palma et al. 2002; Szczykutowicz et al. 2006; Durkin et al. 2015; Leonardi and Quintana 2017; Leonardi et al. 2018).

We have collected lice from Amur Falcons sampled in Nagaland, India at a migratory stopover site, where these birds gather to form huge autumn roosting flocks before crossing the Indian subcontinent and the Arabian Sea towards their African wintering grounds. Our aim was to investigate the effect of falcon sex, age and body size on the abundance of their two most abundant species of lice. We applied generalised linear models to model parasite abundance using host age, wing length and their interaction with sex as explanatory variables.

We demonstrated that the age class (juvenile vs. adult) of Amur Falcons covaries with the abundance of both *C. subzerafae* and *D. rufa* so that juveniles were more infested than adults. Juvenile-biased levels of ectoparasite infestation have been frequently reported by other authors (Potti and Merino 1995; Hamstra and Badyaev 2009; Rivera-Parra et al. 2014), probably due to several reasons. First, juveniles are immunologically naïve, and the amblyceran lice (including *C. subzerafae*) partially feed on living tissues and, thus, interact with the host immune system (Møller and Rózsa 2005). Second, young birds may not be able to allocate as much time to body maintenance behaviours like preening (birds’ primary defence mechanism against lice) as the adult birds. Finally, the efficacy of preening may well depend on individual practice, even though the effect of experience could not be experimentally verified in domestic Rock Pigeons (*Columba livia* Gmelin, 1789; Villa et al. 2016).

The results presented here indicate that *D. rufa* also exhibits female-biased infestation during the autumn period at a migratory stopover site, where the falcons’ habitat usage and behaviour are presumed to be uniform across sexes. Female-biased infestations are not unprecedented; Ortego et al. (2007) showed that the *D. rufa* infestations in Lesser Kestrels (*Falco naumanni* Fleischer, 1818) tend to be female-biased. However, it is far more often the male sex that hosts higher levels of infection (Poulin 1996; Zuk and McKean 1996; Morales-Montor et al. 2004). For example, male-biased louse infestations were found among White-throated Dippers (*Cinclus cinclus* (Linnaeus, 1758); Doyle et al. 2005) and various seabirds (Rivera-Parra et al. 2014).

The causes of female bias observed here are not known, but several possible reasons can be put forward to explain this phenomenon:

First, one could hypothesize that females constitute more optimal hosts for these lice due to their somewhat larger body size. This seems unlikely since we have statistically controlled for body size (using wing length as a proxy for it) in the present study, and also in our other study on Red-footed Falcons (*Falco vespertinus* Linnaeus, 1766; Piross et al. 2020).

Second, Red-footed Falcons — previously considered conspecific with Amur falcons — also exhibit female-biased infestations by the same two louse species examined here, at least during the early days of the breeding period (Piross et al. 2020). During the breeding season, the female-biased infestations might be driven by a difference in how sexes allocate their time; females may allocate less time to body maintenance. The Amur falcons’ female-biased infestations with *D.rufa* during the autumn migration could simply be a remnant of a previous infestation difference that built up during the summer breeding season. However, this does not explain why only *D. rufa* showed female-biased infestation. A notable difference between the two species is that they evade preening differently. *D. rufa* similarly to some other ischnocerans attaches itself to feathers. Amblycerans, on the other hand, tend to use more active evasion techniques and different refugia. *Colpocephalum* species, for example, may hide inside of feather shafts (Clayton and Johnson 2003). Assuming that there is a difference here in the efficacy of the two broad evasion strategies, we hypothesize that the females may be able to reduce the number of *C. subzerafae* more quickly; however, this explanation lacks empirical evidence.

Lastly, female-biased parasitism can be attributed to sexual dichromatism. Adult male Amur Falcons and Red-footed Falcons display a dark bluish-grey colouration based on melanin pigments. Since melanin is known to make feathers more resistant to mechanical abrasion (Bonser 1995), dark feathers may be harder to chew for lice. Contrary to this expectation, however, Bush et al. (2006) showed that dark colouration in rock pigeons provided no defence against the pigeon wing lice, *Columbicola columbae* (Linnaeus, 1758). However, the ischnoceran wing lice like *C. columbae* and *D. rufa* graze the barbules of the down feathers that tend to be unpigmented, but they do not feed on the heavily pigmented, interlocked barbs of the vane of cover feathers (Clayton et al. 2015). Therefore, the difference in the melanin pigmentation between the sexes is unlikely to cause the infestation differences we documented.

Overall, we conclude that birds can show age- and sex-biased levels of louse infestation even during migration, when birds of different age and sex live an apparently similar way of life. Our study highlights the importance of following the seasonal changes of sex-biased parasite infections through the whole life cycle of the host to develop a better understanding of host-parasite systems.

# Acknowledgements

This study was taken up as part of an ongoing project “Understanding the Amur Falcons *Falco amurensis*, their stop-over sites in Nagaland and their migratory routes for better conservation planning”, and funding for this was provided to R.S.K by the Ministry of Environment, Forest and Climate Change (Wildlife Division), Government of India. Permissions and logistic support to undertake this study was provided by Nagaland State Forest Department. R.S.K. would like to specifically thank Sh. Sathya Prakash Tripathy, CWLW Nagaland; Dr V. B. Mathur, Director, and Dr G. S. Rawat, Dean of WII; local people of Pangti, Yaongyimchen and Hakhizhe in Nagaland for their support.

We also would like to thank Lucie Oslejskova and Oldrich Sychra (University of Veterinary and Pharmaceutical Sciences Brno, Brno, Czech Republic) for their help in the identification of the louse specimens; Memorandum of Understanding on the Conservation of Migratory Birds of Prey in Africa and Eurasia (CMS Raptors MoU) Small Grants Programme for BirdLife Hungary to assist R.S.K.

L.R. was supported by the European Regional Development Fund under the project GINOP 2.3.2-15- 2016-00057.

# Author contributions statement

Péter Fehérvári, Péter Palatitz, Péter Borbáth, R. Suresh Kumar gathered the ectoparasite samples. Szablocs Solt gathered morphological data from the birds. Nóra Vili and Nóra Magonyi developed the exact methodology of the molecular sexing of the birds. Manju Siliwal did the molecular sexing of the birds and identified the louse specimens with the help of Zoltán Vas. Imre Sándor Piross and Andrea Harnos analysed the data and prepared the figures. Imre Sándor Piross, Péter Fehérvári, Lajos Rózsa and Andrea Harnos wrote the first draft of the manuscript and all authors commented on previous versions of the manuscript. All authors read and approved the final manuscript.

# Disclosure of potential conflicts of interests

*Multiple affiliations of Imre Sándor Piross:* Imre Sándor Piross started collaborating in this study as a student at the Doctoral School of the University of Veterinary Medicine, Budapest, Hungary. His current workplace (Balaton Limnological Institute, Centre for Ecological Research, Tihany, Hungary) provided time and resources to finish his work on this study and the manuscript.

*Multiple affiliations of Nóra Magonyi:* Nóra Magonyi is a student of the Doctoral School of Biology and Sportbiology, Faculty of Sciences, University of Pécs, Pécs, Hungary, and conducts her work in collaboration with the Conservation Genetics Research Group, Department of Ecology, Institute for Biology, University of Veterinary Medicine, Budapest, Hungary. Both institutions provided resources for her work in this study.

The authors declare that they have no other conflict of interest.

# Compliance with Ethical Standards

The permit for undertaking the study including capture of Amur Falcons at the three roost sites (Permit No. CWL/GEN/96 (Vol-II)/ 1177-85 dated 26.09.2016) was granted by the Nagaland State Forest Department, India.

## Statement on the welfare of animals

All applicable international, national, and/or institutional guidelines for the care and use of animals were followed.

# Tables

Table 1. Descriptive statistics of the louse infestation of the Amur Falcons (*Falco amurensis*) by age, louse species and sex (SD: standard deviation).

Table 2. Abundances (and their 95% C.I.) of the different louse species on Amur Falcons (*Falco amurensis*) predicted by the GLMs.

# Figures

Figure 1 Results of the GLMs modelling the mean abundance of the louse species on the Amur Falcons (*Falco amurensis*). The mean abundance of *Colpocephalum subzerafae* is higher on juveniles than on males. In the case of *Degeeriella rufa* we found interaction between the sex and the age of the birds. The mean abundance of *D. rufa* is similarly high among juveniles for both sexes, while it is higher for adult females than adult males

# References

Auguie B (2017) gridExtra: Miscellaneous Functions for “Grid” Graphics

Bonser RH (1995) Melanin and the abrasion resistance of feathers. The Condor 97:590–591

Brooks ME, Kristensen K, Benthem KJ van, et al (2017) glmmTMB Balances Speed and Flexibility Among Packages for Zero-inflated Generalized Linear Mixed Modeling. R J 9:378–400

Bulgarella M, Palma RL (2017) Coextinction dilemma in the galápagos islands: Can darwin’s finches and their native ectoparasites survive the control of the introduced fly philornis downsi. Insect Conserv Divers 10:193–199

Bush SE, Kim D, Moyer BR, et al (2006) Is melanin a defense against feather-feeding lice? The Auk 123:153–161

Clayton DH, Bush SE, Johnson KP (2015) Coevolution of life on hosts: integrating ecology and history. University of Chicago Press

Clayton DH, Drown DM (2001) Critical evaluation of five methods for quantifying chewing lice (Insecta: Phthiraptera). J Parasitol 87:1291–1301

Clayton DH, Johnson KP (2003) The biology, ecology, and evolution of chewing lice. In: Chewing lice: world checklist and biological overview (Special Publication 24). Illinois Natural History Survey, pp 451–475

Dougherty ER, Carlson CJ, Bueno VM, et al (2016) Paradigms for parasite conservation. Conserv Biol 30:724–733

Doyle U, Crook AC, Smiddy P, O’Halloran J (2005) Feather lice (Mallophaga) of the Irish Dipper Cinclus cinclus hibernicus. Ringing Migr 22:133–137

Durkin ES, Luong LT, Bird J (2015) Mechanisms underlying parasite infection: influence of host body mass and age on chewing louse distribution among brown-headed cowbirds. Parasitol Res 114:4169–4174

Fox J (2018) RcmdrMisc: R Commander Miscellaneous Functions

Fridolfsson A-K, Ellegren H (1999) A Simple and Universal Method for Molecular Sexing of Non-Ratite Birds. J Avian Biol 30:116–121. https://doi.org/10.2307/3677252

Galloway TD, Lamb RJ (2017) Abundance of chewing lice (Phthiraptera: Amblycera and Ischnocera) increases with the body size of their host woodpeckers and sapsuckers (Aves: Piciformes: Picidae). Can Entomol 149:473–481. https://doi.org/10.4039/tce.2017.18

Hamstra TL, Badyaev AV (2009) Comprehensive investigation of ectoparasite community and abundance across life history stages of avian host. J Zool 278:91–99

Kwak ML (2018) Australia’s vanishing fleas (Insecta: Siphonaptera): a case study in methods for the assessment and conservation of threatened flea species. J Insect Conserv 22:545–550

Kwak ML, Heath AC, Palma RL (2019) Saving the Manx Shearwater Flea Ceratophyllus (Emmareus) fionnus (Insecta: Siphonaptera): The Road to Developing a Recovery Plan for a Threatened Ectoparasite. Acta Parasitol 1–8

Lamb RJ, Galloway TD (2016) Seasonal population dynamics of chewing lice (Phthiraptera: Amblycera and Ischnocera) infesting three species of woodpeckers (Aves: Piciformes: Picidae) in Manitoba, Canada. Can Entomol 148:683–692

Lamb RJ, Galloway TD (2019) Host body size and the abundance of chewing lice (Phthiraptera: Amblycera, Ischnocera) infesting eight owl species (Aves: Strigiformes) in Manitoba, Canada. Can Entomol 1–8. https://doi.org/10.4039/tce.2019.43

Lenth RV (2016) Least-squares means: the R package lsmeans. J Stat Softw 69:1–33

Leonardi MS, Quintana F (2017) Lousy chicks: Chewing lice from the Imperial Shag, Leucocarbo atriceps. Int J Parasitol Parasites Wildl 6:229–232

Leonardi MS, Svagelj WS, Gómez Laich A, Quintana F (2018) The eldest sibling is the lousiest in an obligate brood-reducer seabird. Emu-Austral Ornithol 118:212–217

Liébana MS, Santillán MÁ, Cicchino AC, et al (2011) Ectoparasites in free-ranging American Kestrels in Argentina: implications for the transmission of viral diseases. J Raptor Res 45:335–342

Møller AP, Rózsa L (2005) Parasite biodiversity and host defenses: chewing lice and immune response of their avian hosts. Oecologia 142:169–176

Morales-Montor J, Chavarria A, De León MA, et al (2004) Host gender in parasitic infections of mammals: an evaluation of the female host supremacy paradigm. J Parasitol 90:531–546. https://doi.org/10.1645/GE-113R3

Moyer BR, Gardiner DW, Clayton DH (2002) Impact of feather molt on ectoparasites: looks can be deceiving. Oecologia 131:203–210. https://doi.org/10.1007/s00442-002-0877-9

Órdenes JS-M, Ibáñez CB, Contreras LR, et al (2005) Ectoparasitismo en tiuque común Milvago chimango chimango (Vieillot, 1816)(Aves, Falconidae) en la zona de Ñuble, Chile. Lundiana

Ortego J, Aparicio JM, Calabuig G, Cordero PJ (2007) Risk of ectoparasitism and genetic diversity in a wild lesser kestrel population. Mol Ecol 16:3712–3720

Palma RL, Johnson AR, Cezilly F, et al (2002) Diversity and distribution of feather lice on Greater Flamingoes (Phoenicopterus ruber roseus) in the Camargue, southern France. N Z Entomol 25:87–89

Piross IS, Fehérvári P, Vas Z, et al (2015) Louse (Insecta: Phthiraptera) infestations of the Amur Falcon (Falco amurensis) and the Red-footed Falcon. Ornis Hung 23:58–65. https://doi.org/10.1515/orhu-2015-0005

Piross IS, Solt S, Horváth É, et al (2020) Sex-dependent changes in the louse abundance of red-footed falcons (Falco vespertinus). Parasitol Res 119:1327–1335. https://doi.org/10.1007/s00436-020-06634-2

Potti J, Merino S (1995) Louse Loads of Pied Flycatchers: Effects of Host’s Sex, Age, Condition and Relatedness. J Avian Biol 26:203–208. https://doi.org/10.2307/3677320

Poulin R (1996) Sexual Inequalities in Helminth Infections: A Cost of Being a Male? Am Nat 147:287–295. https://doi.org/10.1086/285851

Price RD, Hellenthal RA, Palma RL, et al (2003) The chewing lice: World checklist and biological overview. Illinois Natural History Survey

R Core Team (2019) R: A Language and Environment for Statistical Computing. R Foundation for Statistical Computing, Vienna, Austria

Reiczigel J, Marozzi M, Fábián I, Rózsa L (2019) Biostatistics for Parasitologists – A Primer to Quantitative Parasitology. Trends Parasitol 35:277–281. https://doi.org/10.1016/j.pt.2019.01.003

Rivera-Parra JL, Levin II, Parker PG (2014) Comparative ectoparasite loads of five seabird species in the Galapagos Islands. J Parasitol 100:569–578

Rózsa L (1997) Patterns in the Abundance of Avian Lice (Phthiraptera: Amblycera, Ischnocera). J Avian Biol 28:249–254. https://doi.org/10.2307/3676976

Rózsa L, Vas Z (2015) Co-extinct and critically co-endangered species of parasitic lice, and conservation-induced extinction: should lice be reintroduced to their hosts? Oryx 49:107–110

Saxena AK (2017) Population characteristics of Black Kite lice. J Parasit Dis 41:684–686

Suh A, Kriegs JO, Brosius J, Schmitz J (2011) Retroposon Insertions and the Chronology of Avian Sex Chromosome Evolution. Mol Biol Evol 28:2993–2997. https://doi.org/10.1093/molbev/msr147

Szczykutowicz A, Adamski Z, Hromada M, Tryjanowski P (2006) Patterns in the distribution of avian lice (Phthiraptera: Amblycera, Ischnocera) living on the great grey shrike Lanius excubitor. Parasitol Res 98:507–510

Tinajero R, Chapa-Vargas L, Ham-Dueñas JG, Santiago-Alarcon D (2019) Haemosporidian infection of the American Kestrel in the southern Chihuahua Desert, Mexico: relationship with land use. J Ornithol 160:699–710

Villa SM, Campbell HE, Bush SE, Clayton DH (2016) Does antiparasite behavior improve with experience? An experimental test of the priming hypothesis. Behav Ecol 27:1167–1171

West AG, Waite DW, Deines P, et al (2019) The microbiome in threatened species conservation. Biol Conserv 229:85–98

Wickham H (2016) ggplot2: Elegant Graphics for Data Analysis. Springer-Verlag New York

Yosef R, Strutzer O, Tabibi R, Rózsa L (2019) Infestations of Lice of Steppe Buzzards (Buteo buteo vulpinus) Differ from those of Common Buzzards (Buteo buteo buteo). J Raptor Res 53:102–108

Yunik ME, Waterman JM, Galloway TD (2016) Seasonal changes in the infestation parameters of the sucking louse, Linognathoides laeviusculus (Phthiraptera: Anoplura: Polyplacidae), infesting Richardson’s ground squirrel (Rodentia: Sciuridae) in Manitoba, Canada. Can Entomol 148:143–150

Zuk M, McKean KA (1996) Sex differences in parasite infections: Patterns and processes. Int J Parasitol 26:1009–1024. https://doi.org/10.1016/S0020-7519(96)80001-4

Zuur A, Ieno EN, Walker N, et al (2009) Mixed Effects Models and Extensions in Ecology with R. Springer-Verlag, New York
